# Genotoxic and cytotoxic potential of whole plant extracts of Kalanchoe laciniata by Ames and MTT assay

**DOI:** 10.17179/excli2016-748

**Published:** 2017-04-24

**Authors:** Ali Sharif, Muhammad Furqan Akhtar, Bushra Akhtar, Ammara Saleem, Maria Manan, Maryam Shabbir, Muneeb Ashraf, Sohaib Peerzada, Shoaib Ahmed, Moosa Raza

**Affiliations:** 1 Faculty of Pharmacy, the University of Lahore, Lahore, Pakistan; 2 Institute of Pharmacy, Physiology and Pharmacology, University of Agriculture, Faisalabad, Pakistan; 3 Faculty of Pharmaceutical Sciences, GC University, Faisalabad, Pakistan; 4 Postgraduate Medical Institute, Jail Road, Lahore, Pakistan

**Keywords:** Kalanchoe laciniata, mutagenicity, cytotoxicity, ames assay, Salmonella typhimurium

## Abstract

Lack of data on safety of herbal medicines have endangered human health and life. The present study evaluated the genotoxic and mutagenic effect of *Kalanchoe laciniata* to access the safety and usefulness of the medicinal plant. Aqua-methanolic and n-hexane extracts of *K. laciniata *were evaluated for the genotoxic potential using Ames assay and cytotoxicity was evaluated using MTT assay. Ames assay was conducted using two strains of *Salmonella typhimurium *TA-100 and TA-102 whereas MTT assay was performed on baby hamster kidney cell line BHK-21. Aqua-methanolic extract of *K.* *laciniata* exhibited significant mutagenicity when exposed to TA-102 strain with a mutagenic index of 50.66 and 54.74 at maximum dose 150 mg/plate. The extract was also mutagenic to TA-100 strain but to a lesser extent. M.I of n-hexane extract was 12.15 and 15.51 for TA-100 and TA-102 respectively. n-hexane extract was mutagenic but little difference was observed between results of two strains. Both extracts were found to be cytotoxic with an IC_50_ of 321.9 and 638.5 µg/mL for aqua-methanolic and n-hexane extracts respectively. On the basis of results it was concluded that aqua-methanolic and n-hexane extracts of *K.* *laciniata* possess mutagenic and cytotoxic potential. It is suggested to explore the plant further to evaluate its safety in rodents and other species.

## Introduction

Plants have been used in alternative and traditional medicines for the cure of different types of diseases. Contemporary medicine has been developed from traditional folkloric system followed by extensive pharmacological and chemical characterization (Akhtar et al., 2016[1]; Ncube et al., 2008[21]). Medicinal plants are considered, the best bio-sources not only for traditional medicine but also for various food supplements, nutraceuticals and synthetic drugs (Boopathi and Sivakumar, 2013[11]). It has been estimated that world's 80 % population relied upon the use of herbal medicines for the remedy of diseases. WHO has issued the directives to encourage the practice of herbal medicines to address health related issues only if non toxicity of the preparations have been established (WHO, 2002[27]; Akintonwa et al., 2009[6]).

*Kalanchoe laciniata* belongs to the family Crassulaceae. It is commonly known as Patharchat or well renowned Christmas tree. The plant is found in tropical regions such as certain parts of Yemen, India, Brazil, Burma, and certain African countries (Joshi et al., 1992[15]). This species has been used as folk medicine for the treatment of diabetes, gastric discomfort, ulcerative sores, diarrhea and dysentery. The study of phytochemical constituents provided the scientific basis about the chemical nature of plants which is found useful in further biological screening. It was reported previously that* K. laciniata *has saponins, tannins, terpenoids, flavonoids, glycosides and anthraquinones (Manan et al., 2015[18]).

Although certain therapeutic advantages of *K. laciniata *have been reported (Manan et al., 2016[19]), it was an established fact that certain constituents of medicinal plants have toxic potential and induce carcinogenic and teratogenic influences (Gadano et al., 2006[14]; Akinboro and Bakare, 2007[5]). 

*In vitro* toxicity testing has gained significant importance as a tool for the evaluation of safety of different drugs and chemicals. The compound which exhibits a mutagenic activity does possess the carcinogenic potential (Boada et al., 2016[9]). Breakage of DNA strands or alteration in the nitrogenous bases is a domineering characteristic of certain chemicals. These alterations lead to uncontrolled proliferation, carcinogenesis or alteration of phenotypes. Ames assay is a reliable tool to access short term toxicity owing to its ability to detect mutagens of different origins (Sharif et al., 2016[23]). 

MTT assay is a calometric assay and is considered a valid tool for the determination of cytotoxic potential of various chemicals, drugs, environmental pollutants and plant extracts (Boncler et al., 2014[10]). This *in vitro* test determines the viability of cells on exposure to toxicants and serves as an indicator of anti-proliferative activation, cell activation and cytotoxicity (Khasawneh et al., 2011[16]). The principle of the test is based on the measurement of formazan color which is formed by the enzymes present in the mitochondria of living cells (Stockert et al., 2012[24]).

The present study focuses on the investigation of mutagenic and cytotoxic potential of aqua-methanolic and n-hexane extracts of whole plant *K. laciniata*. 

## Material and Methods

The plant used in this study was *K. laciniata *(Crassulaceae). The plant was selected on the basis of above mentioned ethnobotanical uses and availability. Fresh plant was collected from different regions of Faisalabad. It was later identified from Dr. Mansoor Hameed, University of Agriculture, Faisalabad. It was shade dried at 25 °C. The dried plant material was ground into coarse powder using an herbal grinder in the lab. 

### Chemicals and reagents

The chemicals used in the study were obtained from Sigma Aldrich^® ^and Fisher Scientific^®^*. *Both the tester strains of *Salmonella typhimurium* TA-100 and TA-102 along with S9 activation mixture were obtained from Environmental Bio-Detection Products Incorporation, Canada.

### Preparation of extracts

Extraction was carried out using two solvent systems. Aqua-methanolic (30-70 %) and n-hexane extracts were prepared using cold maceration. After one week period the extracts were filtered using a Whatsmann filter paper and filtrate was subjected to dry in rotary evaporator. The dried extracts were weighed and collected in glass vials. 

### Mutagenic activity

Bacteria was used as an experimental model to identify the mutagenic potential of two extracts of *K. laciniata *whole plant. Ames pre-incubation assay was conducted using *Salmonella typhimurium* (His^-^) strains TA-100 and TA-102 alone and in combination with S9 activation mixture. *S. typhimurium *TA100 and TA102 strains were obtained in freeze dried form. They were processed in a biological safety cabinet (Class II) and poured into 1.0 mL of nutrient broth previously sterilized to ensure that the bacterial growth was in log phase. One colony collected from purified culture of both strains was poured into 5 mL of nutrient broth. 24 hours incubation period was given at 37 °C. The cell growth was confirmed by streaking these cultures on pre-sterilized nutrient agar plates which were incubated overnight. Mutant tester strains were inoculated in sterilized nutrient broth 15-18 hours prior to perform experiment and fresh cultures of bacteria were obtained in exponential growing (log) phase (Mortelmans and Zeiger, 2000[20]). Each plate was provided with equal number of bacterial (2×10^8^) colonies. 0.1 mL suspension of both strains of *S. typhimurium* TA-100 and TA-102 were poured into autoclaved test tubes containing 0.5 mL of test extract dilutions. Two fold dilutions were prepared starting from 150 mg/plate to 9.375 mg/plate. The mixture was vortexed to mix thoroughly and an incubation period of 20 minutes was given. Test tube contents were poured onto the GM agar plates containing Vogel-Boner medium and incubated at 37 °C for 48 hours. The experiment was performed with and without metabolic activation mixture in which 0.5 mL of enzyme liver fraction was also added in test tube along with bacteria and test extract. All the experiments were conducted in triplicate. The plates were observed for the presence of revertant colonies under a colony counter which have a characteristic appearance of pin point dew drops. Sodium azide (Na-Azide) was used as positive control without S9 activation mixture and 2-aminoanthracycline (2-AA) was used as the positive control when S9 activation mixture was incorporated (Sharif et al., 2016[23]). Mutagenic Index (MI) was calculated using the formula in which number of revertant colonies of the test extracts were divided by the number of revertant colonies of the negative control. Sample was considered mutagenic if the MI of the tested concentration was greater than 2 (Akhtar et al., 2016[2]). 

### MTT assay

Baby hamster kidney cell lines (BHK-21), kindly provided by Quality Operations Laboratory, UVAS Lahore, were cultured in DMEM which is augmented with fetal bovine serum 20 %. The cells were placed in CO_2_ incubator at 37 °C to attain a confluent monolayer. Trypsin (0.25 % EDTA) was used to detach the cells from the flask and again sub-cultured. MTT assay which is a calorimetric analysis was used to determine the cell viability. The principle of the assay is reduction of MTT dye (3-(4, 5-dimethylthiazol-2-yl)-2, 5-diphenyltetrazolium bromide) due to the dehydrogenase enzyme present in metabolically active cells. MTT dye enters into the mitochondria and convertes into a purple color formazan crystal which is insoluble. Later cells were solubilized and quantified using a spectrophotometer (Akram et al., 2013[7]). The level of metabolic activity is directly proportional to viability of cells as only viable cells have the ability to reduce tetrazolium dye. The MTT assay was conducted using a 96 well flat bottom cell culture plate and confluent layer of BHK-21 was seeded into the wells. These cells were permitted to adhere and placed overnight in an incubator and later every dilution of the extract was exposed to these cells. Dilutions of the extract were prepared in DMEM and incubated for 48 hours. Viability of BHK-21 cells was determined by adding 20 µL of MTT dye solution (50 mg/10 mL) in each well and giving an incubation period of 3 hours (Akhtar et al., 2016[3]). DMSO was used to lyse the cells and optical densities were measured at 570 nm with ELISA reader. The cell survival percentage (CSP) was measured with the help of the following formula

Cell survival percentage (CSP) = = (Mean optical density of test chemical - Mean Optical density of negative control × 100) / Mean Optical density of positive control.

### Statistical analysis

The number of revertant colonies evaluated after Ames assay were expressed as mean and Standard deviation, further regression analysis was applied to determine the slope values and ensuring dose-dependent behavior. Cell survival percentage was calculated using regression analysis and IC_50_ was calculated using non-linear fit of equation. The data was analyzed using graph pad prism 6 and MS Word/Excel 2013 software.

## Results

### Mutagenicity

**Figure 1:**
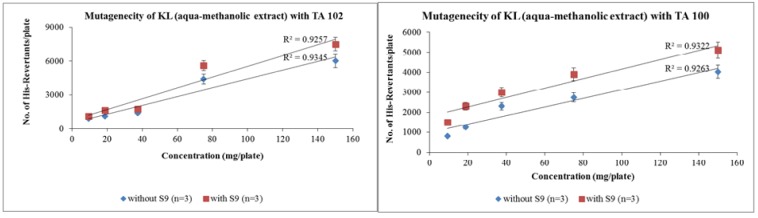
Dose-dependent increase in revertant colonies of aqua-methanolic extract of *Kalanchoe laciniata* whole plant with Salmonella typhimurium TA 102 and TA 100 strains

The number of revertant colonies decreased with the increase in dilution factor. Aqua-methanolic extract exhibited maximum number of colonies when exposed to TA-102 strain. The number of colonies increased from 6051 ± 31 to 7519 ± 17 when enzyme activation mixture was introduced. Maximum number of revertant colonies appeared at maximum concentration of 150 mg/plate. However, a significant reduction in the number of revertant colonies was observed when the dose was decreased to 9.375 mg/plate. Pattern of colony induction in the presence of enzyme activation mixture was same. At a dose of 9.375 mg/plate the number of revertant colonies increased from 520 ± 17 to 734 ± 27 due to enzyme induction. Figure 1(Fig. 1) depicts the increase in the number of revertant colonies in aqua-methanolic extract when treated with both tester strains. In both cases enzyme induction increased the number of resultant colonies at all doses tested.

**Figure 2:**
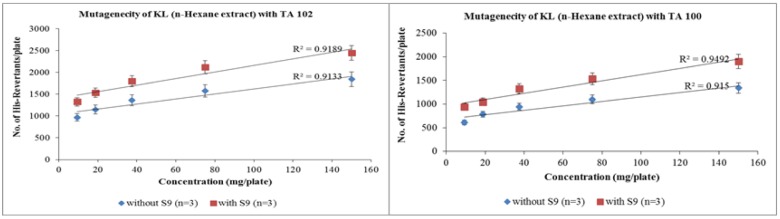
Dose-dependent increase in revertant colonies of n-hexane extract of *Kalanchoe laciniata* whole plant with *Salmonella typhimurium* TA 102 and TA 100 strains

Similar pattern was followed when n-hexane extracts were treated with both tester strains. However, the slope values obtained from regression analysis were much lower than that of aqua-methanolic extracts. The revertant colonies of n-hexane extracts with TA-100 were raised from 417 ± 6 to 625 ± 2.64 when exposed to metabolic activation mixture strain at dose of 9.375 mg/mL. At maximum dose of 150 mg/mL the number of revertant conies were 1845 ± 2 and 2366 ± 4 in absence and presence of activation mixture respectively. Figure 2(Fig. 2) shows that the number of revertant colonies increased with increase in concentration of n-hexane extract. It is also evident that activation mixture was able to increase the number the revertant colonies in both cases.

Mutagenic index was calculated in each case by dividing the number of revertant colonies of the test dose of both extracts with the number of revertant colonies of the negative control. It was found that the lowest concentrations of both extracts were significantly mutagenic even in the absence of activation mixture. The lowest concentration of both extracts, that is 9.375 mg/plate, was proved to be significantly mutagenic. The values of mutagenic indices were given in Tables 1(Tab. 1) and 2(Tab. 2) for both extracts in the absence and presence of activation mixture.

### Cytotoxicity

Cell survival percentage for both extracts of *K. laciniata *increased in a dose-dependent manner as is evident from Tables 3(Tab. 3) and 4(Tab. 4). Cell survival percentage (CSP) at which growth of 50 % of BHK-21 cells got inhibited was calculated using graph pad prism software. IC_50_ of aqua-methanolic and n-hexane extracts were 321.9 and 638.5 µg/mL respectively.

**Figure 3:**
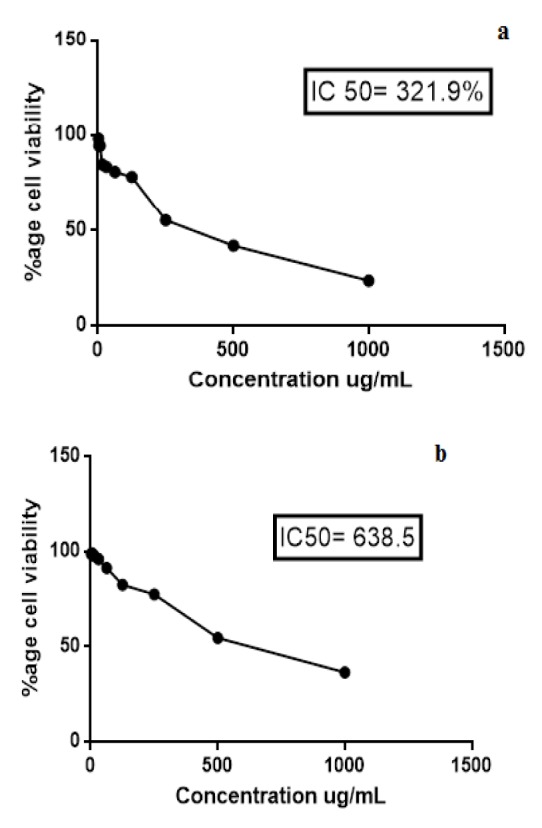
Cytotoxicity assay of (a) aqua-methanolic and (b) n-hexane extracts of *Kalanchoe laciniata* whole plant extracts

The aqua-methanolic extract retained more anti-proliferative activity than n-hexane extract. Figure 3(Fig. 3) depicts the IC_50_ of aqua-methanolic and n-hexane extracts of *Kalanchoe laciniata* whole plant.

Statistical evaluation of data revealed that the number of revertant colonies increase with the increase in concentration. Slope values *m* for aqua-methanolic *K. laciniata *whole plant extracts with and without S9 were 39.25 and 48.06 for TA-102 strain whereas 24.01 and 28.97 were slope values obtained for TA 100 strain. Similarly slope values for n-hexane extracts of *K. laciniata *whole plants were 5.79 and 6.38 for TA-102 and 5.76 and 8.08 for TA-100 with and without metabolic activation mixture respectively. The mutagenic index also decreased in dose-dependent fashion. Cell viability was increased with the decrease in concentration of extract.

For more results see Supplementary data.

## Discussion

A dose-dependent mutagenic effect was observed in both extracts when treated with TA-100 and TA-102 strains of *S. typhimurium*. The number of revertant colonies increased with the increase in concentration of extracts. The pattern is followed by both extracts. Results obtained from regression analysis demonstrates steeper slope values for TA-102 as compared to TA-100 indicating that aqua-methanolic extract responded more when treated with TA-102 as compared to TA-100 (Sharif et al., 2016[23]). However, in both cases the number of revertant colonies increased when samples were exposed to metabolic activation mixture suggesting that plant extracts have a greater potential to induce DNA damage when under goes metabolism. Slopes for n-hexane extract are shallower when compared to aqua-methanolic extract exhibiting minor damage. This little damage may be attributed to lesser soluble components in n-hexane extract owing to its nonpolar nature. However, exposing the n-hexane extract to metabolic activation mixture does follow the previous pattern and an increase in the number of revertant colonies was observed due to enzyme induction. The plant extracts were highly mutagenic. Mutagenic index decreased in a dose-dependent fashion. MI of aqua-methanolic extract was higher with TA-102 suggesting that the mechanism of DNA damage was transition induced. These results are in accordance with previous studies (Akhtar et al., 2016[4]). Oxidizing agents have a potential to induce transition mutations leading to oxidative stress (Degtyareva et al., 2013[12]). The plant extracts also showed mutagenicity when exposed to TA-100 strain suggesting that base pair mutation is also the possible mechanism of mutagenicity. The results exhibited a mutagenic response in both extracts, however, a previous study on another species of the same genus reported that the juice of plant was not mutagenic which is in contrast with this study (Umbuzeiro-Valent et al., 1999[26]). Reactive oxygen species and constituents possessing oxidizing properties are linked to membrane damage and carcinogenic potential. Antioxidant use is recommended for the prevention of diseases and improvement of human health (Del Rio et al., 2013[13]). 

Cytotoxic activity of *K. laciniata *was not reported previously but constituents of *Kalanchoe pinnata* which include five bufadienolides were investigated and their anti-proliferative activity was confirmed (Supratman et al., 2001[25]). A dose-dependent cytotoxic fashion was observed in the study suggesting a probable therapeutic utility of this medicinal plant. Earlier studies by Biswas et al. (2011[8]) demonstrated presence of similar cytotoxic activity against brine shrimp *Artemia salina* in ethanolic extract of *Kalanchoe pinnata. *It was investigated that aqua-methanolic and n-hexane extracts of *K. laciniata *were cytotoxic to BHK-21 cell line with IC_50_ of 638.5 and 321.9 µg/mL respectively. IC_50_ values of both extracts were much higher than *Kalanchoe pinnata* n-hexane extract which exhibited IC_50_ of 75.7 μg/mL against hormone-dependent breast cancer (MCF-7) carcinoma cell lines and 100 μg/mL against colon cancer (Caco2) cell lines (Rahmat et al., 2005[22]).

Preliminary screening using MTT assay results revealed, aqua-methanolic and n-hexane extracts of *Kalanchoe pinnata* possesses anti-proliferative and cytotoxic potential. Crude leaf extracts of *B. pinnata,* a plant with same species but different genera, exhibited an IC_50_ of 552 µg/mL (Mahata et al., 2012[17]).

## Conclusion

The present study concludes that *Kalanchoe laciniata* whole plant extracts were significantly mutagenic and do exhibit cytotoxic potential. Both genotoxicity and cytotoxicity was aggravated with the increase in concentration of the aqua-methanolic and n-hexane extracts. Based upon the results of this *in vitro* investigation, it is referred that *K. laciniata* is not considered to be safe for use in humans. We propose, deeper investigations must be performed to access the safety of *K. laciniata* whole plant extracts using animal models to access for any pathological lesions and alteration in biochemical parameters.

## Conflict of interest

The authors have no conflict of interest.

## Supplementary Material

Supplementary data

## Figures and Tables

**Table 1:**
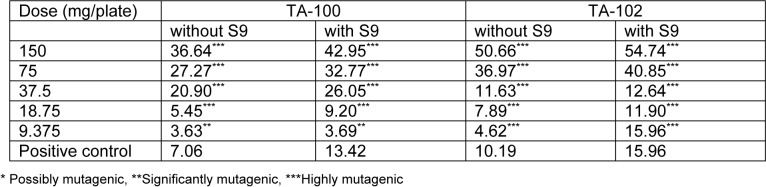
Mutagenic index of different doses of *Kalanchoe laciniata* whole plant aqua-methanolic extract in absence and presence of metabolic activation mixture S9 on exposure to two strains of *Salmonella typhimurium*

**Table 2:**
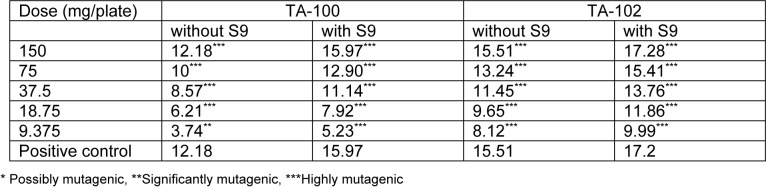
Mutagenic index of different doses of *Kalanchoe laciniata* whole plant n-hexane extract in absence and presence of metabolic activation mixture S9 on exposure to two strains of *Salmonella typhimurium*

**Table 3:**
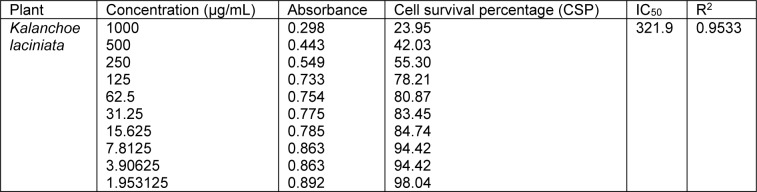
Cell survival percentage (CSP) increase with the decrease in concentration of aqua-methanolic extracts of *Kalanchoe laciniata* whole plant

**Table 4:**
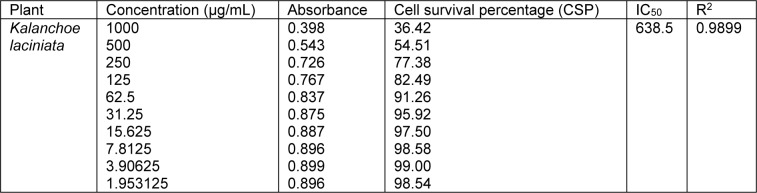
Cell survival percentage (CSP) increase with the decrease in concentration of n-hexane extracts of *Kalanchoe laciniata* whole plant
