# Supplementary material for: Genotoxic and cytotoxic potential of whole plant extracts of Kalanchoe laciniata by Ames and MTT assay
Source: EXCLI J. 2017 Apr 24;16:593–601. doi: 10.17179/excli2016-748 (PMC5491922; doi:10.17179/excli2016-748)
Supplement: Supplementary data [file EXCLI-16-593-s-001.pdf]

EXCLI Journal 2017;16:593-601 – ISSN 1611-2156
Received: November 03, 2016, accepted: April 03, 2017, published: April 24, 2017

Supplementary data to:

GENOTOXIC AND CYTOTOXIC POTENTIAL OF WHOLE PLANT EXTRACTS OF KALANCHOE LACINIATA BY AMES AND MTT ASSAY

Ali Sharif[1*], Muhammad Furqan Akhtar[1], Bushra Akhtar[2], Ammara Saleem[3], Maria Manan[3], Maryam Shabbir[1], Muneeb Ashraf[4], Sohaib Peerzada[1], Shoaib Ahmed[1], Moosa Raza[1]

[1] Faculty of Pharmacy, the University of Lahore, Lahore, Pakistan
[2] Institute of Pharmacy, Physiology and Pharmacology, University of Agriculture, Faisalabad, Pakistan
[3] Faculty of Pharmaceutical Sciences, GC University, Faisalabad, Pakistan
[4] Postgraduate Medical Institute, Jail Road, Lahore, Pakistan

* Corresponding author: Ali Sharif, Faculty of Pharmacy, the University of Lahore, Off Defense Road Lahore. E-mail: alisharif.pharmacist@gmail.com

http://dx.doi.org/10.17179/excli2016-748

This is an Open Access article distributed under the terms of the Creative Commons Attribution License (http://creativecommons.org/licenses/by/4.0/).

EXCLI Journal 2017;16:593-601 – ISSN 1611-2156
Received: November 03, 2016, accepted: April 03, 2017, published: April 24, 2017

MUTAGENIC INDEX OF AQUA-METHANOLIC EXTRACT

Table 1: Number of revertant colonies used in calculations

**Number of revertant colonies per plate**				
**Concentration (mg/plate)**	TA-100 (no. of revertant colonies) without S9	TA-100 (no. of revertant colonies) with S9	TA-102 (no. of revertant colonies) without S9	TA-102 (no. of revertant colonies) with S9
**150**	4031	5112	6029	7500
**75**	3000	3900	4400	5597
**37.5**	2300	3100	1384	1732
**18.75**	600	1101	940	1631
**9.375**	400	440	550	1110
**Positive control**	777	1597	1213	2187
**Negative Control**	110	119	119	137

Table 2: Mutagenic Index of respective dilutions

**Mutagenic Index of respective dilutions calculated as M.I. =(number of revertant colonies of respective concentration/number of revertant colonies of negative control)**				
	TA-100 (no. of revertant colonies)	TA-100 (no. of revertant colonies)	TA-102 (no. of revertant colonies)	TA-102 (no. of revertant colonies)
**Concentration (mg/plate)**	Mutagenic Index (without S9)	Mutagenic Index (with S9)	Mutagenic Index (without S9)	Mutagenic Index (with S9)
**150**	36.64545455	42.95798	50.66387	54.74453
**75**	27.27272727	32.77311	36.97479	40.85401
**37.5**	20.90909091	26.05042	11.63025	12.64234
**18.75**	5.454545455	9.252101	7.89916	11.90511
**9.375**	3.636363636	3.697479	4.621849	15.9635
**Positive Control**	7.063636364	13.42017	10.19328	15.9635

Table 3: Mean of number of revertant colonies

**Concentration (mg/plate)**	**TA 100 (without S9)**	**TA 100 (with S9)**	**TA 100 (without S9)**	**TA 102 (with S9)**
**150**	4031.333333	5112	6029	7500
**75**	3000	3933.333333	4400	5596.667
**37.5**	2300	3100	1384	1732.333
**18.75**	600	1101	940	1633.333
**9.375**	400	440	550	1110
**Positive Control**	777	1597	1213	2186.667

EXCLI Journal 2017;16:593-601 – ISSN 1611-2156
Received: November 03, 2016, accepted: April 03, 2017, published: April 24, 2017

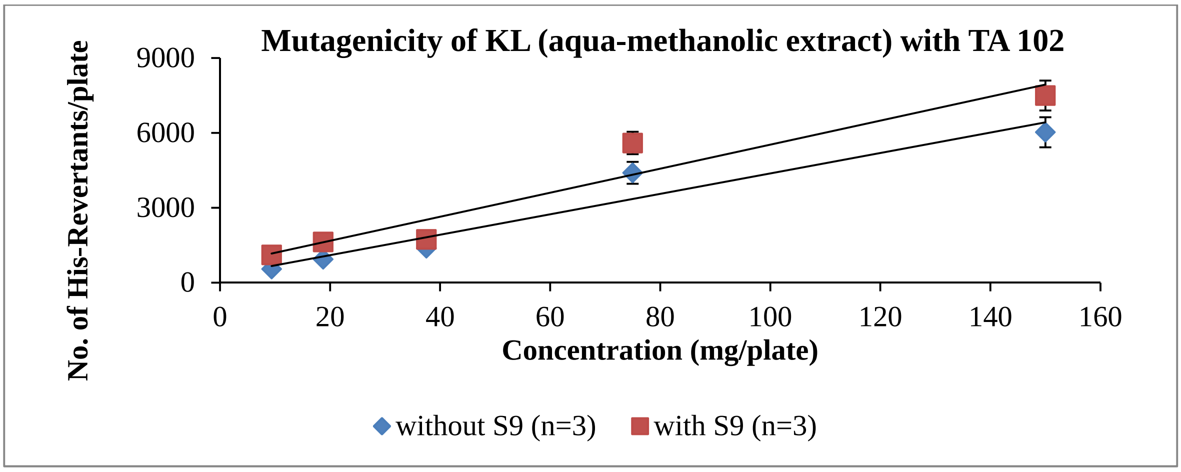

Figure 1: Dose-dependent increase in revertant colonies of aqua-methanolic extract of *Kalanchoe laciniata* whole plant with *Salmonella typhimurium* TA 102 strain

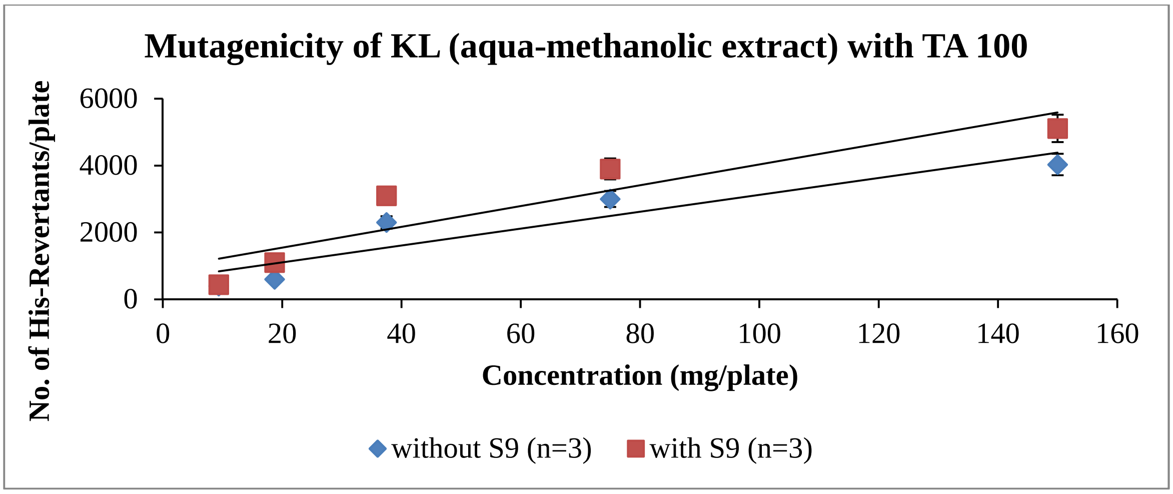

Figure 2: Dose-dependent increase in revertant colonies of aqua-methanolic extract of *Kalanchoe laciniata* whole plant with *Salmonella typhimurium* TA 100 strains

EXCLI Journal 2017;16:593-601 – ISSN 1611-2156
Received: November 03, 2016, accepted: April 03, 2017, published: April 24, 2017

MUTAGENIC INDEX OF N-HEXANE EXTRACT

Table 4: Number of revertant colonies used in calculations

Number of revertant colonies per plate				
**Concentration (mg/plate)**	TA-100 (no. of revertant colonies)	TA-100 (no. of revertant colonies)	TA-102 (no. of revertant colonies)	TA-102 (no. of revertant colonies)
	without S9	with S9	without S9	with S9
**150**	1845	2428	1813	2368
**75**	1571	1961	1576	2112
**37.5**	1303	1694	1363	1886
**18.75**	944	1205	1149	1625
**9.375**	569	795	967	1369
**Positive control**	1852	2428	1846	2357
**Negative Control (Distilled water)**	152	152	119	137

Table 5: Mutagenic Index of respective dilutions

Mutagenic Index of respective dilutions calculated as M.I =(number of revertant colonies of respective concentration/number of revertant colonies of negative control)				
**Concenteration (mg/plate)**	TA-100 (no. of revertant colonies)	TA-100 (no. of revertant colonies)	TA-102 (no. of revertant colonies)	TA-102 (no. of revertant colonies)
	without S9	with S9	without S9	with S9
**150**	12.13816	15.97368	15.23529	17.28467
**75**	10.33553	12.90132	13.2437	15.41606
**37.5**	8.572368	11.14474	11.45378	13.76642
**18.75**	6.210526	7.927632	9.655462	11.86131
**9.375**	3.743421	5.230263	8.12605	9.992701
**Positive control**	12.18421	15.97368	15.51261	17.20438
**Negative Control**	152	152	119	137

Table 6: Mean of number of revertant colonies

Concenteration (mg/plate)	TA-100 (without S9)	TA-100 (with S9)	TA-102 (without S9)	TA-102 (with S9)
**150**	1845	2428	1813	2368
**75**	1571	1961	1576	2112
**37.5**	1303	1694	1364	1886
**18.75**	944	1205	1149	1625
**9.375**	569	795	967	1369
**Positive Control**	1852	2428	1846	2357

EXCLI Journal 2017;16:593-601 – ISSN 1611-2156
Received: November 03, 2016, accepted: April 03, 2017, published: April 24, 2017

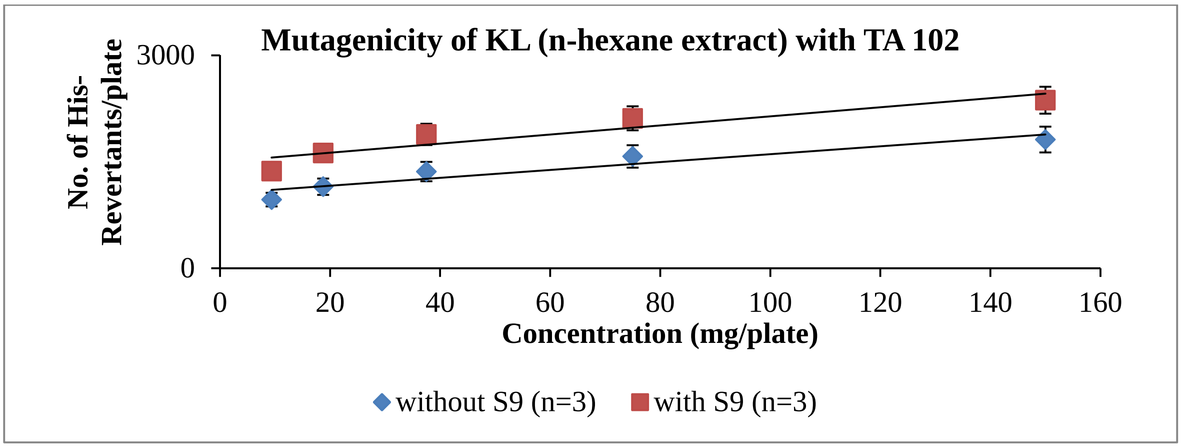

Figure 3: Dose-dependent increase in revertant colonies of n-hexane extract of *Kalanchoe laciniata* whole plant with *Salmonella typhimurium* TA 102 strain

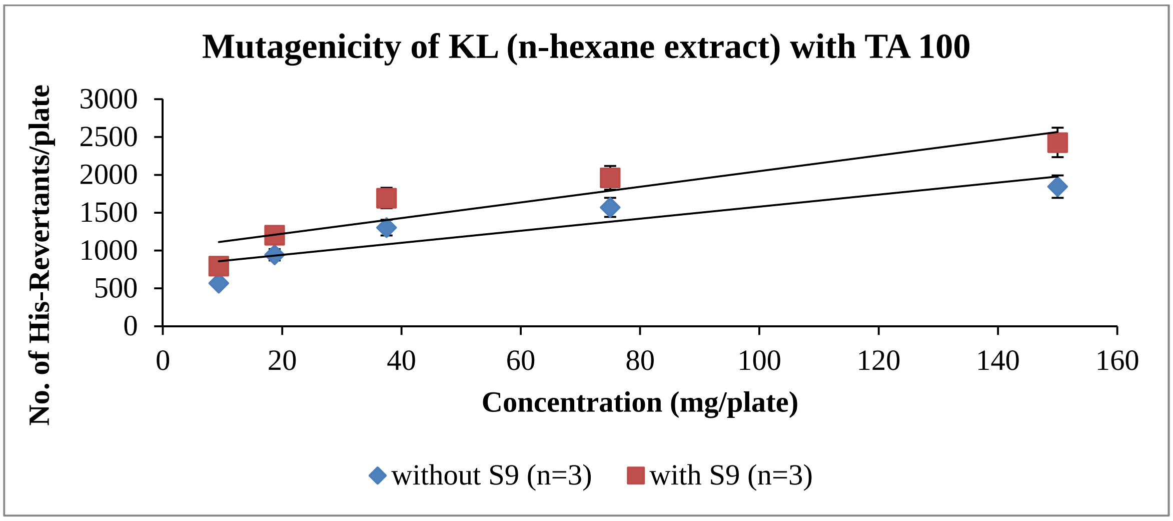

Figure 4: Dose-dependent increase in revertant colonies of n-hexane extract of *Kalanchoe laciniata* whole plant with *Salmonella typhimurium* TA 100 strain

EXCLI Journal 2017;16:593-601 – ISSN 1611-2156
Received: November 03, 2016, accepted: April 03, 2017, published: April 24, 2017

INHIBITORY CONCENTRATION OF AQUA-METHANOLIC EXTRACT CALCULATED FROM GRAPH PAD PRISM

Table 7: Concentration versus percentage viability of aqua-methanolic extracts

Concentration (µg/mL)	Log Concentration (µg/mL)	percentage cell viability
**1000.000000**	3.000	23.95010
**500.000000**	2.69897	42.03742
**250.000000**	2.39794	55.30146
**125.000000**	2.09691	78.21206
**62.500000**	1.79588	80.87318
**31.250000**	1.49485	83.45114
**15.625000**	1.19382	84.74012
**7.812500**	0.89279	94.42827
**3.906250**	0.59176	94.42827
**1.953125**	0.29073	98.04574

RESULTS OF GRAPH PAD PRISM: LOG (INHIBITOR) VS. NORMALIZED RESPONSE

log(inhibitor) vs. normalized response	
Best-fit values	
$LogIC_{50}$	2.508
IC_{50}	321.9
Std. Error	
$LogIC_{50}$	0.04975
95% Confidence Intervals	
$LogIC_{50}$	2.395 to 2.620
IC_{50}	248.4 to 417.1
Goodness of Fit	
Degrees of Freedom	9
R square	0.9533
Absolute Sum of Squares	259.3
Sy.x	5.368
Number of points	
Analyzed	10

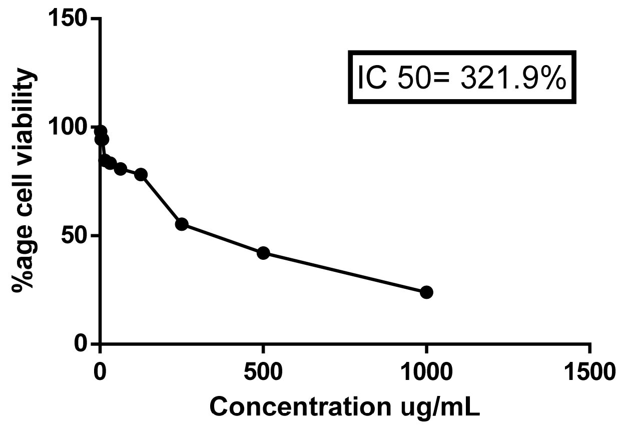

Figure 5: Concentration versus percentage viability of aqua-methanolic extract

EXCLI Journal 2017;16:593-601 – ISSN 1611-2156
Received: November 03, 2016, accepted: April 03, 2017, published: April 24, 2017

INHIBITORY CONCENTRATION OF N-HEXANE EXTRACT CALCULATED FROM GRAPH PAD PRISM

Table 8: Concentration versus percentage viability of n-hexane extracts

Concentration (µg/mL)	Log Concentration (µg/mL)	percentage cell viability
**1000.000000**	3.000	36.42412
**500.000000**	2.69897	54.51143
**250.000000**	2.39794	77.38046
**125.000000**	2.09691	82.49480
**62.500000**	1.79588	91.26819
**31.250000**	1.49485	95.92516
**15.625000**	1.19382	97.50520
**7.812500**	0.89279	98.58628
**3.906250**	0.59176	99.00208
**1.953125**	0.29073	98.54470

RESULTS OF GRAPH PAD PRISM: LOG (INHIBITOR) VS. NORMALIZED RESPONSE

log(inhibitor) vs. normalized response	
Best-fit values	
$LogIC_{50}$	2.805
IC_{50}	638.5
Std. Error	
$LogIC_{50}$	0.02189
95% Confidence Intervals	
$LogIC_{50}$	2.756 to 2.855
IC_{50}	569.7 to 715.6
Goodness of Fit	
Degrees of Freedom	9
R square	0.9899
Absolute Sum of Squares	42.58
Sy.x	2.175
Number of points	
Analyzed	10

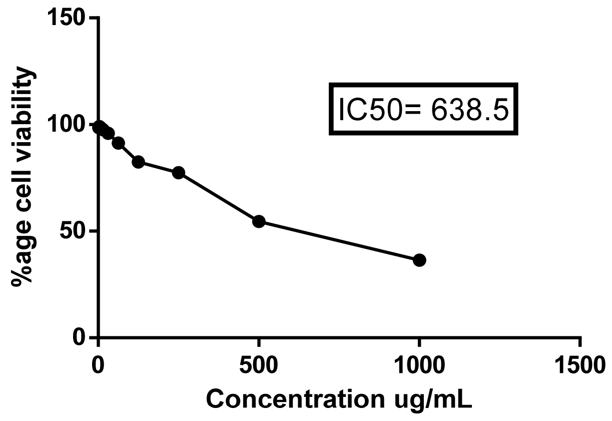

Figure 6: Concentration versus percentage viability of n-hexane extract
